# Sunitinib enhances the antitumor responses of agonistic CD40-antibody by reducing MDSCs and synergistically improving endothelial activation and T-cell recruitment

**DOI:** 10.18632/oncotarget.10364

**Published:** 2016-07-01

**Authors:** Luuk van Hooren, Maria Georganaki, Hua Huang, Sara M. Mangsbo, Anna Dimberg

**Affiliations:** ^1^ Department of Immunology, Genetics and Pathology, Science for Life Laboratory, The Rudbeck Laboratory, Uppsala University, Uppsala, Sweden

**Keywords:** CD40, sunitinib, MDSC, endothelial activation, T-cell

## Abstract

CD40-activating immunotherapy has potent antitumor effects due to its ability to activate dendritic cells and induce cytotoxic T-cell responses. However, its efficacy is limited by immunosuppressive cells in the tumor and by endothelial anergy inhibiting recruitment of T-cells. Here, we show that combining agonistic CD40 monoclonal antibody (mAb) therapy with vascular targeting using the tyrosine kinase inhibitor sunitinib decreased tumor growth and improved survival in B16.F10 melanoma and T241 fibrosarcoma. Treatment of tumor-bearing mice with anti-CD40 mAb led to increased activation of CD11c^+^ dendritic cells in the tumor draining lymph node, while sunitinib treatment reduced vessel density and decreased accumulation of CD11b^+^Gr1^+^ myeloid derived suppressor cells. The expression of ICAM-1 and VCAM-1 adhesion molecules was up-regulated on tumor endothelial cells only when anti-CD40 mAb treatment was combined with sunitinib. This was associated with enhanced intratumoral infiltration of CD8^+^ cytotoxic T-cells. Our results show that combining CD40-stimulating immunotherapy with sunitinib treatment exerts potent complementary antitumor effects mediated by dendritic cell activation, a reduction in myeloid derived suppressor cells and increased endothelial activation, resulting in enhanced recruitment of cytotoxic T-cells.

## INTRODUCTION

Tumors evade destruction by the immune system through secretion of immunosuppressive cytokines, recruitment of immunosuppressive cells, altering the antigen-presentation machinery, up-regulation of immune regulatory molecules and induction of endothelial anergy towards effector immune cell recruitment [1]. By specifically targeting molecules involved in regulation of immune cell function in the tumor microenvironment, the immune response toward the tumor can be activated.

CD40 is a member of the tumor necrosis factor (TNF) superfamily that has attracted considerable interest in the field of immunotherapy of cancer due to its pivotal role in activation of dendritic cells (DCs) and stimulation of adaptive immunity. CD40-activating immunotherapy has shown promising results, and several clinical grade anti-CD40 monoclonal antibodies are being tested in clinical trials [NCT02379741, NCT02482168, NCT02376699, 2-4]. CD40 is expressed on a wide variety of cells, including DCs, macrophages, monocytes, B cells, endothelial cells and some tumor cells, but the main therapeutic activity is considered to be through the licensing of DCs to activate an antitumor cytotoxic T-cell response [5–7]. However, CD40 stimulation has also been implicated in promoting tumor growth and immune evasion. For example, CD40 stimulation may result in increased expression of VEGF and thereby promoting tumor angiogenesis [8], and CD40 signaling on myeloid-derived suppressor cells (MDSCs) is required for induction of T-cell tolerance and the accumulation of T regulatory cells (Tregs) in the tumor [9].

The tumor vasculature acts as a barrier to T-cell recruitment, limiting the efficacy of immunotherapy [10]. Pro-angiogenic factors in the tumor microenvironment induce pathological angiogenesis, which results in tortuous, abnormal vessels with decreased pericyte coverage and poor perfusion [11]. Angiogenic growth factors, including vascular endothelial growth factor (VEGF), dampen the endothelial response to pro-inflammatory signals and inhibit up-regulation of adhesion molecules necessary for recruitment of T-cells to the tumor [12, 13]. Anti-angiogenic treatment can reverse this effect, normalize vessel function and improve leukocyte recruitment [14]. We have demonstrated that VEGF-stimulation inhibits TNF-α-induced nuclear factor ĸ B (NF-ĸB) signaling in endothelial cells, leading to reduced up-regulation of pro-inflammatory genes including T-cell attracting chemokines CXCL10 and CXCL11 [15]. Inhibition of VEGFR-signaling by sunitinib treatment in B16.F10 melanoma bearing mice leads to enhanced expression of chemokines and adhesion molecules on tumor endothelial cells and a higher number of CD3^+^ T-cells in the tumor [15]. Sunitinib is a tyrosine kinase inhibitor that acts on a broad range of receptor tyrosine kinases including VEGFR, PDGFR, c-Kit and FLT3 [16]. In addition to its anti-angiogenic properties, sunitinib treatment reduces the number of immunosuppressive MDSCs and regulatory T-cells (Tregs) in the tumor and decreases expression of CTLA-4 and PD-1 on tumor infiltrating T-cells [17]. Given the multifunctional effects of sunitinib on the vasculature and on leukocytes, there is a potential for mechanistically complementary effects with immunotherapy [17, 18].

Here, we have investigated the potential benefit of combining agonistic anti-CD40 antibody therapy with sunitinib treatment in B16.F10 melanoma and T241 fibrosarcoma. These syngeneic experimental models of cancer have been used in immunotherapeutic preclinical settings and are characterized by an abnormal vessel phenotype and neovascularization. We find that in addition to relieving endothelial anergy and improving T-cell recruitment, sunitinib reverses CD40-induced MDSC accumulation in the lymph node, leading to smaller tumors and improved survival when the treatments are combined as compared to either therapy alone.

## RESULTS

### Combining local agonistic anti-CD40 antibody therapy with sunitinib treatment reduces B16.F10 melanoma and T241 fibrosarcoma tumor growth and prolongs survival

**Figure 1:**
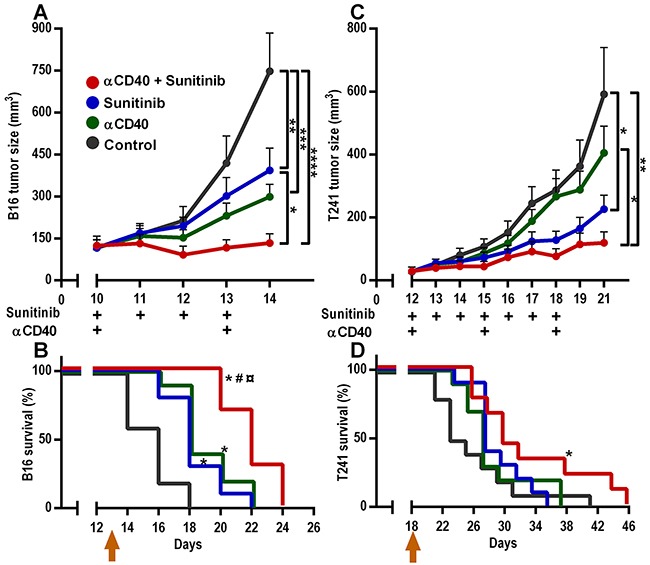
Combined anti-CD40 mAb and sunitinib therapy reduces B16.F10 melanoma and T241 fibrosarcoma tumor growth and improves survival **A.** B16.F10 melanoma tumor growth (n=10, mean, SEM, *p < 0.05, **p < 0.01, ***p < 0.001, ****p < 0.0001, one-way ANOVA, the graph shows one representative experiment out of five independent experiments). **B.** B16.F10 melanoma Kaplan-Meier survival curve (n= 10, *p < 0.05 vs control, ^#^*p* < 0.05 vs anti-CD40 mAb, ^¤^*p* < 0.05 vs sunitinib, Gehan-Breslow-Wilcoxon test, the graph shows one representative survival experiment out of two independent experiments.) **C.** T241 fibrosarcoma tumor growth (n=9-10, mean, SEM, *p < 0.05, **p < 0.01, one-way ANOVA, the graph shows one representative experiment out of three independent experiments.) **D.** T241 fibrosarcoma Kaplan-Meier survival curve (n=9-10, *p < 0.05 vs control, Gehan-Breslow-Wilcoxon test). Arrows in B and D indicate the last day of treatment.

To evaluate the therapeutic efficacy of combining agonistic anti-CD40 monoclonal antibody (anti-CD40 mAb) therapy with sunitinib treatment, we analyzed the response to combination therapy or monotherapy in two different murine subcutaneous tumor models, B16.F10 melanoma and T241 fibrosarcoma. The treatment started when the tumors were palpable. We have previously shown that a local low-dose of anti-CD40 mAb is superior to systemic treatment, and that four days of sunitinib treatment is sufficient to induce chemokine expression in B16.F10 tumor endothelial cells [15, 19]. Therefore, anti-CD40 mAb therapy was administrated locally on day 10 and 13 after tumor injection, and sunitinib was given by oral gavage on days 10-13. Treatment with either anti-CD40 mAb or sunitinib alone had a moderate effect on B16.F10 tumors, while the combination of anti-CD40 mAb therapy and sunitinib substantially decreased tumor growth (Figure 1A). Consistent with this, combining anti-CD40 mAb therapy with sunitinib treatment resulted in a significant survival benefit in comparison to both monotherapies and the control group (Figure 1B, Supplementary Figure S1A-S1D). T241 fibrosarcoma tumors have a slower growth pattern than B16.F10 melanoma. Anti-CD40 mAb therapy was therefore given locally on day 12, day 15 and day 18, and sunitinib was given for 7 consecutive days (day 12-18). Despite high CD40 expression (Supplementary Figure S4B), anti-CD40 mAb therapy alone did not inhibit T241 tumor growth, while there was a trend towards reduced tumor size after sunitinib treatment (Figure 1C). Combining anti-CD40 mAb therapy with sunitinib significantly inhibited tumor growth and increased survival (Figure 1C-1D, Supplementary Figure S1E-S1H). These results show that the combination of sunitinib and anti-CD40 mAb is superior to either monotherapy in B16.F10 melanoma and T241 fibrosarcoma.

### CD40-induced activation of DCs is not inhibited by sunitinib co-treatment

**Figure 2:**
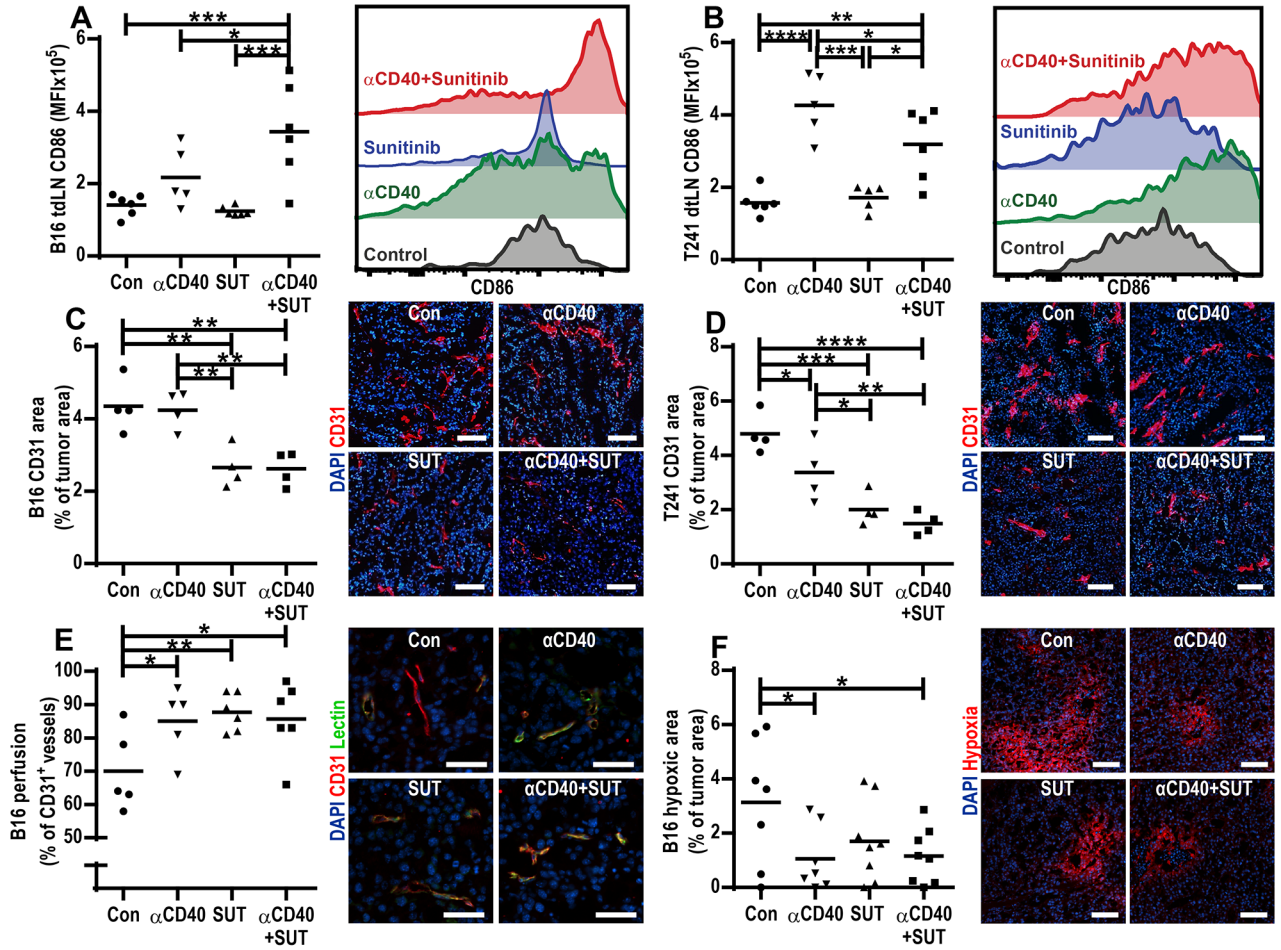
Effects of anti-CD40 mAb, sunitinib treatment and the combination therapy on activation of cDCs, tumor vessel density, vessel function and tumor hypoxia **A-B.** Surface expression of CD86 on cDCs (B220^−^CD11b^+/−^CD11c^+^) in the tumor draining lymph nodes of B16.F10 (A) and T241 (B) tumor bearing mice measured by flow cytometry. Graphs show mean fluorescence intensity (MFI), and data points represent analysis of tumor draining lymph nodes in different mice one day after the last treatment (Mean, *p < 0.05, **p < 0.01, ***p < 0.001, ****p < 0.0001, one-way ANOVA). The histograms are representative examples of CD86 fluorescence intensity for each treatment group. **C-D.** Immunofluorescence CD31 staining of vessels in B16.F10 (C) and T241 (D) tumors and vessel density quantifications (CD31-positive area/tumor area, mean, *p < 0.05, **p < 0.01, ***p < 0.001, ****p < 0.0001, one-way ANOVA). Data points represent analysis of entire tumor sections in different mice, representative images of tumors in each treatment group are shown (CD31-red, Hoechst 33342- blue, 10 × magnifications, scale bar: 100 μm). **E.** Percentage of CD31^+^ vessels perfused with biotin-labeled lycopersicon esculentum (tomato) lectin in B16.F10 tumors. Each data point indicates the percentage of perfused vessels per tumor (5 optical fields, 20x, mean, *p < 0.05, **p < 0.01, one-way ANOVA). Representative microscopic images for each treatment group are shown (20x, scale bar: 25 μm, CD31-red, lectin-green, Hoechst 33342-blue). **F.** Percentage of hypoxic area in B16.F10 tumors (5 optical fields, 10x magnification, mean, *p < 0.05, one-way ANOVA) and representative images for each treatment group (10x, scale bar: 100 μm, Hypoxic area-red, Hoechst-blue). The analysis of tumor draining lymph nodes and tumors were performed one day after the last treatment.

Stimulation of CD40 by anti-CD40 mAb activates DCs, licensing them to induce an antitumor cytotoxic T lymphocyte response [6]. Conversely, inhibition of VEGF-signaling during DC maturation *in vitro* or by treating tumor-bearing mice with sunitinib *in vivo* has previously been demonstrated to inhibit DC activation [20, 21]. Therefore, we investigated if combining anti-CD40 mAb therapy with sunitinib treatment affects the activation status of conventional DCs (cDCs) (B220^−^CD11b^+/−^CD11c^+^) in the tumor draining lymph node. This was performed by measuring the surface expression of CD86, a co-stimulatory molecule required for T-cell activation by DC [22]. In the B16.F10 melanoma model, CD86 surface expression was significantly up-regulated on cDCs in tumor draining lymph nodes in mice treated with anti-CD40 mAb in combination with sunitinib versus control (Figure 2A). In the T241 fibrosarcoma model, increased surface expression of CD86 on cDCs was observed in the tumor draining lymph nodes of mice treated with anti-CD40 mAb alone and in mice treated with anti-CD40 mAb in combination with sunitinib (Figure 2B). Sunitinib treatment alone did not alter CD86 expression on DCs.

### Sunitinib treatment reduces vessel density in B16.F10 and T241 tumors

CD40-stimulation has been shown to induce VEGF-expression, and thereby stimulate angiogenesis [8], while sunitinib inhibits angiogenesis in several types of tumors [23]. We evaluated if treatment either with anti-CD40 mAb, sunitinib or the combination therapy affects vessel density by performing immunostaining of the endothelial marker CD31 in sections from B16.F10 and T241 tumors. In both B16.F10 and T241 tumors, treatment with sunitinib, alone or in combination with anti-CD40 mAb, reduced vessel density (Figure 2C-2D). Treatment with anti-CD40 mAb alone decreased vessel density in the T241 model, although to a lesser extent than sunitinib therapy (Figure 2D). Co-stainings of CD31 and desmin showed that the tumor vessel pericyte coverage was similar in all treatment groups in both tumor models (Supplementary Figure S2A-S2B). To determine if either anti-CD40 or sunitinib treatment affected vessel function, we injected biotin-labeled lectin and Hypoxyprobe-1 (pimonidazole hydrochloride) in B16.F10 tumor-bearing mice. Vascular perfusion was enhanced by treatment with anti-CD40 mAb and/or sunitinib (Figure 2E), and tissue hypoxia was decreased (Figure 2F).

### The CD40-induced increase of CD11b^+^Gr1^+^ cells in the tumor draining lymph node is reduced by sunitinib co-treatment

**Figure 3:**
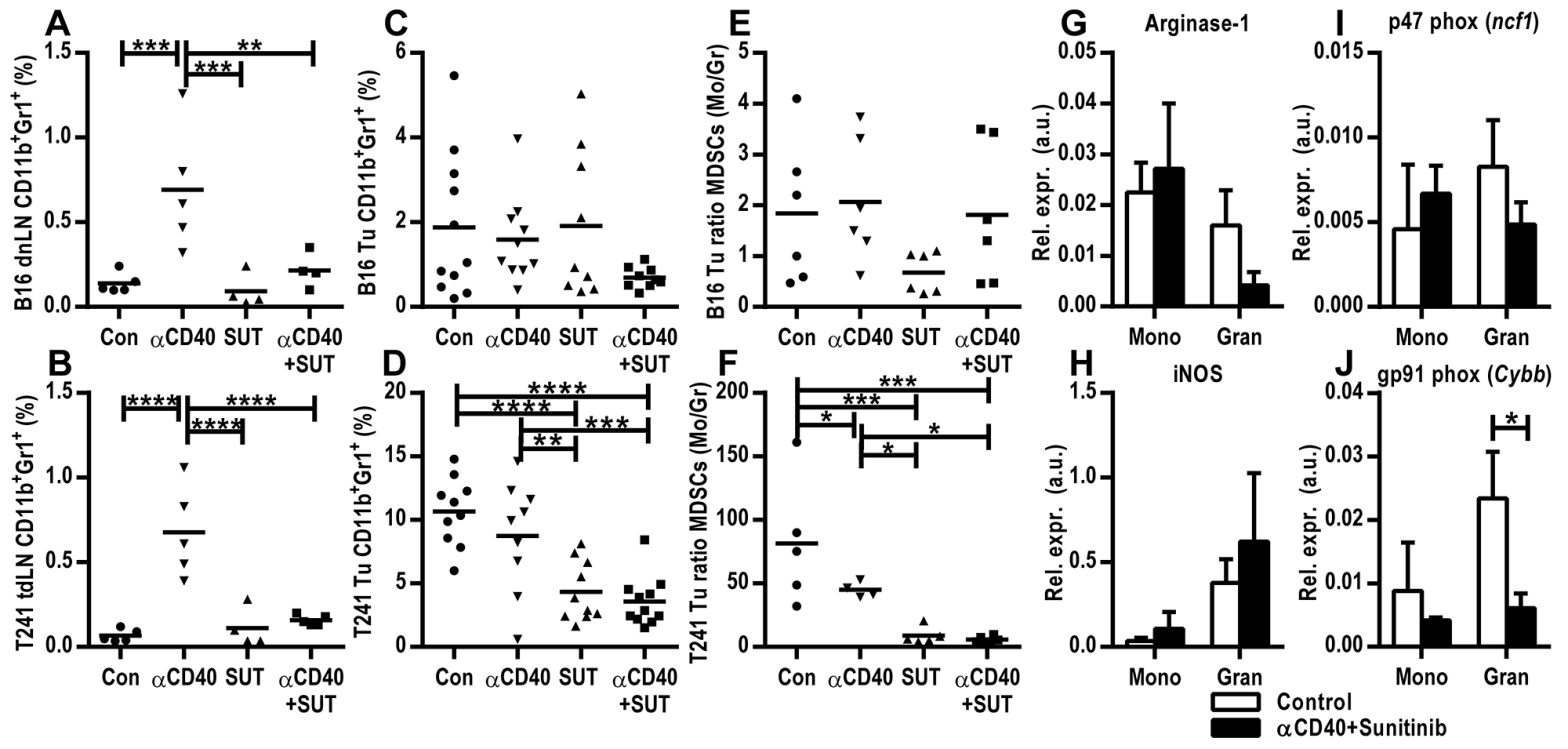
Sunitinib treatment reduces the CD11b^+^Gr1^+^ myeloid-derived suppressor cell population in the tumor and tumor draining lymph node **A.** FACS analysis of tumor draining lymph nodes from mice bearing B16.F10 tumors. **B.** FACS analysis of tumor draining lymph nodes from mice bearing T241 tumors. Data from one representative experiment out of two independent experiments are shown. **C-D.** FACS analysis of B16.F10 (C) and T241 tumors (D). Merged data from two experiments are shown. The values indicate the percentages of CD11b^+^Gr1^+^ MDSCs of the total number of cells as measured by flow cytometry (mean, *p < 0.05, **p < 0.01, ***p < 0.001, ****p < 0.0001, one-way ANOVA). **E-F.** Ratios of monocytic to granulocytic MDSCs in B16.F10 (E) and T241 (F) tumors (mean, *p < 0.05, **p < 0.01, ***p < 0.001, one-way ANOVA). Data points represent analysis of tumor draining lymph nodes in different mice one day after the last treatment. **G-J.** TaqMan qPCR analysis of arginase-1 (G), iNos (H), p47phox (I) and gp91phox (J) gene expression in FACS-sorted monocytic and granulocytic MDSCs isolated from B16.F10 tumors. Values depict expression relative to actin (n=3 tumors from different mice, mean, SD, *p < 0.05, unpaired Student's t-test). FACS-sorting was performed two days after the last treatment.

CD11b^+^Gr1^+^ MDSCs contribute to the immunosuppressive tumor milieu by suppressing T-cells, and have been implicated in refractoriness to anti-VEGF therapy [24, 25]. We sought to examine how this cell population is affected by the combined anti-CD40 mAb and sunitinib therapy in the tumor and peripheral lymphoid tissue. Anti-CD40 mAb treatment increased the percentage of CD11b^+^Gr1^+^ cells in the tumor draining lymph node in both the B16.F10 and T241 model, but co-treatment with sunitinib reversed the effect (Figure 3A-3B). In the B16.F10 tumors, there was a strong trend towards a decrease of CD11b^+^Gr1^+^ MDSCs in the tumor tissue in the combination therapy group (p=0.058, Figure 3C). In the T241 tumors there was a significant decrease of CD11b^+^Gr1^+^MDSCs when sunitinib was given alone or in combination with anti-CD40 mAb therapy (Figure 3D). These results demonstrate that co-treatment with sunitinib can reverse the increase in CD11b^+^Gr1^+^ cells caused by agonistic anti-CD40 antibody treatment in the tumor draining lymph node, while the effect on tumor CD11b^+^Gr1^+^ MDSCs is dependent on the tumor model.

Murine MDSCs are subcategorized as monocytic or granulocytic reflecting the cellular origin. The monocytic MDSCs (Mo-MDSCs) are characterized as CD11b^+^Ly6G^lo^Ly6C^+^ and the granulocytic (Gr-MDSCs) as CD11b^+^Ly6G^+^Ly6C^+^ [26, 27]. Since we observed a difference in the total MDSC population in the tumors treated with agonistic anti-CD40 antibodies in combination with sunitinib, we sought to identify if this was correlated with an altered ratio of Mo-MDSCs to Gr-MDSCs. In the B16.F10 model the ratio and percentage of Mo-MDSCs to Gr-MDSCs in tumor tissue was similar in all groups (Figure 3E, Supplementary Figure S3A-S3B). In the T241 model the ratio of Mo-MDSCs to Gr-MDSCs was significantly decreased when sunitinib was administrated alone or in combination with anti-CD40 mAb (Figure 3F). This was due to a decrease of the monocytic subset (Supplementary Figure S3C-S3D), which has previously been suggested to be the most important immunosuppressive subset *in vivo* [26]. FACS-isolated monocytic and granulocytic MDSCs from B16.F10 tumors treated with either the combination of agonistic-CD40 antibody and sunitinib or control were subjected to qPCR analysis of a set of genes that have previously been associated with immunosuppression [27, 28]. Expression of arginase 1, an enzyme associated with T-cell suppression, and iNOS (inducible nitric oxide synthase), which mediates NO production, was found in both Mo-MDSCs and Gr-MDSCs (Figure 3G, 3H). Similarly, both Mo-MDSCs and Gr-MDSCs expressed the P47phox and gp91phox (NOX2) subunits of the NADPH oxidase 2 involved in ROS production (Figure 3I-3J). We conclude that the monocytic and granulocytic MDSCs isolated from tumors from control and anti-CD40 mAb/sunitinib-treated mice express enzymes that can mediate immunosuppression.

### Sunitinib and anti-CD40 mAb therapy synergistically induce endothelial activation

**Figure 4:**
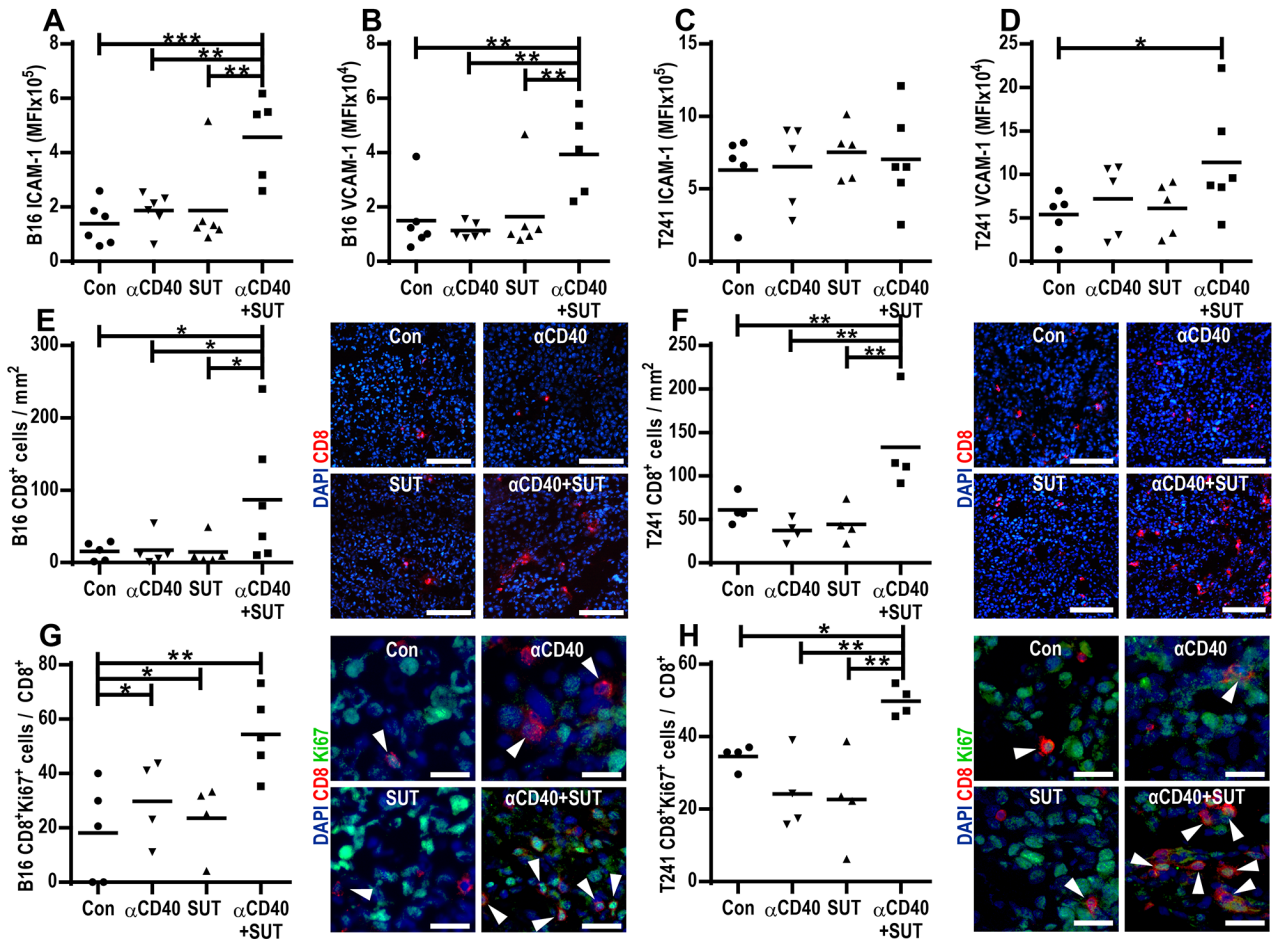
Anti-CD40 and sunitinib therapy synergistically induce endothelial activation and cytotoxic CD8^+^ T-cell infiltration into the tumor **A-D.** Endothelial activation in B16.F10 and T241 tumors treated with anti-CD40 mAb and/or sunitinib or control. FACS analysis of ICAM-1 and VCAM-1 surface expression in endothelial cells (CD45^−^CD31^+^) from B16.F10 (A-B) and T241 tumors **C-D.** Values represent MFI of ICAM-1 and VCAM-1 in tumor endothelial cells isolated from different mice (mean, *p < 0.05, **p < 0.01, ***p < 0.001, one-way ANOVA). **E-F.** Quantification of CD8^+^ (red) T-cell immunofluorescence staining in B16.F10 (E) and T241 (F) tumor sections counterstained with Hoechst 33342 (blue). The graphs show the number of CD8^+^ cells/mm^2^ in tumor sections from different mice (mean, *p < 0.05, **p < 0.01, one-way ANOVA). Representative images from B16.F10 (E) and T241 (F) tumor sections are shown (20x, scale bar: 100 μm) **G-H.** Ratio of proliferating Ki- 67^+^CD8^+^ to total CD8^+^ T-cells in B16.F10 (G) and T241 (H) tumors analyzed by immunofluorescence staining (data points show analysis of 5 optical fields/tumor, mean, *p < 0.05, **p < 0.01, one-way ANOVA) and representative images (40x, scale bar: 25 μm, Ki-67-green, CD8-red, Hoechst 33342-blue). Data points indicate analysis of tumors in different mice one day after the last treatment.

To investigate if combining anti-CD40 mAb therapy with sunitinib treatment affects tumor endothelial cell activation, we studied the surface expression of the intercellular adhesion molecule 1 (ICAM-1) and the vascular cell adhesion molecule 1 (VCAM-1) in B16.F10 and T241 tumor-derived endothelial cells. In the B16.F10 melanoma model, endothelial expression of ICAM-1 and VCAM-1 was not changed after treatment with agonistic CD40-antibody or sunitinib alone, but combining these treatments led to a synergistic increase in ICAM-1 (p=0.043, Figure 4A) and VCAM-1 (p=0.017, Figure 4B) surface expression. In the T241 fibrosarcoma model, there was a high expression of ICAM-1 and VCAM-1 in the tumor vasculature in control mice. No further increase of ICAM-1 expression was observed. However, similar to the B16.F10 melanoma model, there was a significant increase in endothelial expression of VCAM-1 for the group treated with anti-CD40 mAb in combination with sunitinib (Figure 4C-4D). These data show that combining anti-CD40 mAb with sunitinib treatment increases the expression of endothelial adhesion molecules and thereby potentially facilitates leukocyte recruitment. We have previously demonstrated that VEGF-induced signaling interferes with NF-kB signaling, leading to down-regulation of pro-inflammatory genes, and that sunitinib treatment can reverse these effects [15]. Consistent with VEGF-stimulation of tumor endothelial cells being associated with lower expression of endothelial activation markers, B16.F10 tumors express higher levels of VEGF and lower levels of endothelial ICAM-1 and VCAM-1 than T241 tumors (Figure 4A-4D, Supplementary Figure S4A). Our results suggest that combining anti-CD40 mAb therapy with sunitinib treatment can synergistically increase expression of endothelial adhesion molecules in tumor vessels that have a repressed expression of these molecules due to VEGF stimulation.

### Combining anti-CD40 mAb therapy with sunitinib treatment enhances infiltration of cytotoxic CD8^+^ T-cells

Similar to several other immunotherapeutic approaches, anti-CD40 mAb therapy elicits antitumor effects mainly through inducing T-cell immune responses. To determine how treatment with anti-CD40 mAb, sunitinib or the combination affects the prevalence of cytotoxic T-cells within the tumor tissue, we performed immunostaining for the cytotoxic T-cell marker CD8 in B16.F10 and T241 tumor sections. There were no significant changes in the prevalence of CD8^+^ T-cells for the groups treated with anti-CD40 mAb or sunitinib alone as compared to control tumors. However, the number of intratumoral CD8^+^ T-cells was increased when mice were treated with the combination of agonistic CD40 antibodies and sunitinib (Figure 4E-4F). This striking effect was observed for both models and was most pronounced in the T241 fibrosarcoma model, resulting in a synergistic increase (p=0.004) in tumor infiltrating CD8^+^ T-cells. The proportion of CD8^+^ T-cells that were proliferating was significantly higher in B16.F10 and T241 tumors from mice treated with the combination of anti-CD40 mAb and sunitinib (Figure 4G-4H). A smaller percentage of CD8^+^ T-cells co-expressed granzyme B in all treatment groups, with a trend towards higher expression in tumors treated with anti-CD40 mAb in combination with sunitinib (Supplementary Figure S5A-S5B). Taken together, these data show that combined treatment with anti-CD40 mAb and sunitinib leads to enhanced infiltration of cytotoxic T-cells in B16.F10 and T241 tumors.

### Continued treatment with anti-CD40 mAb in combination with sunitinib further enhances survival of mice bearing B16.F10 melanoma

**Figure 5:**
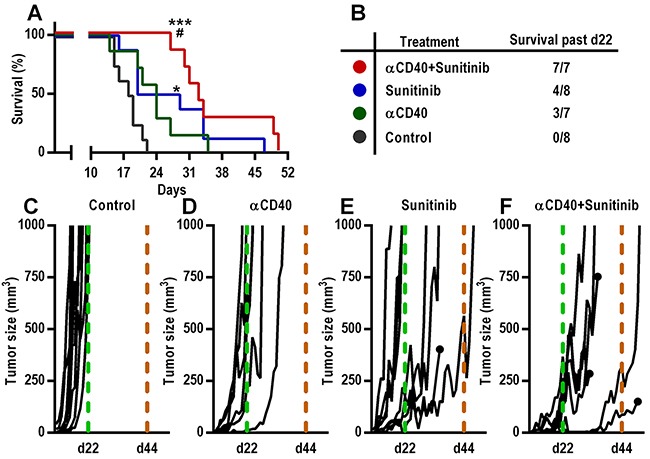
Increased survival of mice bearing B16.F10 melanoma tumors after continued treatment with anti-CD40 mAb in combination with sunitinib **A-B.** Kaplan-Meier survival curve (A) of B16.F10 tumor bearing mice continually treated with agonistic CD40-antibodies (90 μg) and sunitinib and corresponding table (B) demonstrating the number of surviving mice beyond day 22 after tumor inoculation (n=7-8, *p < 0.05 vs control, ***p < 0.001 vs control, ^#^*p* < 0.05 vs αCD40, Gehan-Breslow-Wilcoxon test). **C-F.** Tumor growth curves of all individual mice from each treatment group. Day 22 (all mice from control group sacrificed) and 44 (last day of treatment) are indicated with a dashed light grey and a dashed black line respectively. The growth curves from mice that were sacrificed for reasons other than tumor size ≥ 1 cm^3^ (health status parameters) are indicated with a black dot.

To determine the efficacy of long-term combination therapy of anti-CD40 mAb and sunitinib, we treated B16.F10 tumor-bearing mice with the combination, the monotherapies or control for 5 weeks (35 days) and sacrificed mice when the tumors reached a volume of 1 cm^3^. Prolonged treatment with anti-CD40 mAb (90 μg) in combination with sunitinib led to an improved survival versus anti-CD40 mAb monotherapy or control (Figure 5A). Importantly, all mice treated with anti-CD40 mAb in combination with sunitinib lived beyond day 22, as compared to 0/8 control mice, 3/7 mice treated with anti-CD40 mAb alone and 4/8 mice treated with sunitinib alone (Figure 5B-5F). Continued treatment with sunitinib as a monotherapy inhibited tumor growth only when the tumor size at the start of the treatment was smaller than 20 mm^3^, but was not effective in larger tumors (Figure 5E), which is in agreement with previous reports [29–31]. These results show that continued treatment with anti-CD40 mAb in combination with sunitinib is superior to monotherapies and further enhances the survival of mice bearing B16.F10 melanomas.

## DISCUSSION

**Figure 6:**
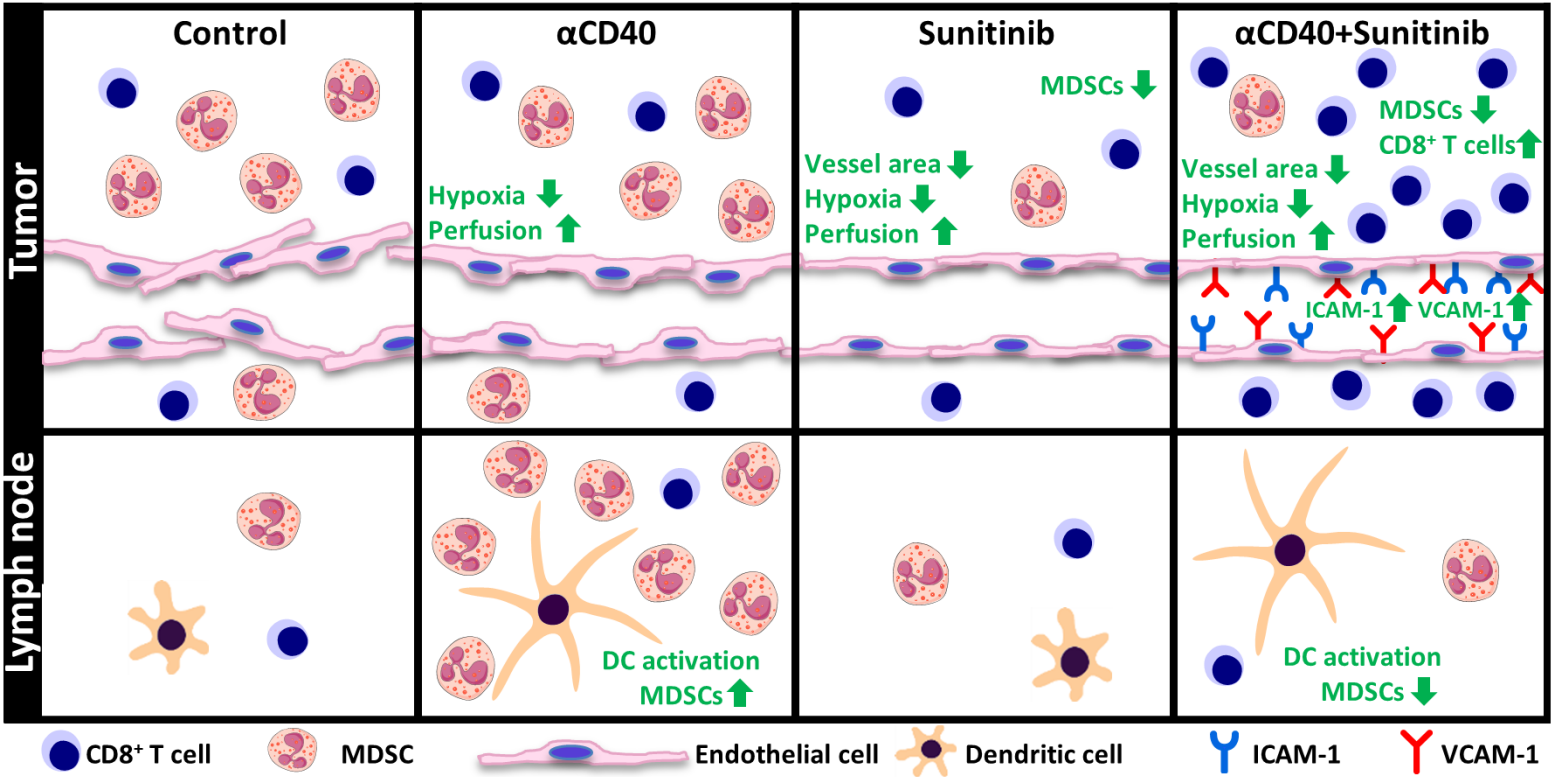
Schematic representation of the combined effects of agonistic anti-CD40 mAb treatment and sunitinib or individual monotherapies in the tumor and tumor draining lymph node In the tumor draining lymph node, anti-CD40 mAb treatment, either alone or in combination with sunitinib, induces activation of cDCs. Treatment with anti-CD40 mAb leads to accumulation of MDSCs in the tumor draining lymph node, an effect which is reversed by sunitinib. In the tumor, improved vessel function and reduced hypoxia was observed for all treatment groups as compared to control. Combining anti-CD40 mAb with sunitinib causes a synergistic increase in endothelial activation and enhanced cytotoxic CD8^+^ T-cell infiltration.

Agonistic anti-CD40 antibody represents a new class of immunotherapeutic drugs, inducing T-cell activation through licencing of DCs in the tumor microenvironment and in the tumor draining lymph nodes. Our previous observation that sunitinib can enhance CD3^+^ T-cell recruitment in the tumor microenvironment through increased chemokine expression suggested that sunitinib co-treatment may enhance the efficacy of cancer immunotherapy [15]. Sunitinib is a multi-targeted tyrosine kinase inhibitor which is approved for the treatment of several cancer types, including renal cell carcinoma (RCC), gastrointestinal stromal tumors and pancreatic neuroendocrine tumors. However, although sunitinib is effective in the initial setting, resistance mechanisms arise during the course of therapy [32]. Our data herein demonstrate that combining CD40-activating immunotherapy with sunitinib treatment enhances DC activation, decreases the number of MDSCs and induces endothelial activation, leading to increased infiltration of cytotoxic T-cells into tumor tissue and reduced tumor growth (Figure 6).

VEGF is one of the most potent pro-angiogenic factors expressed by human tumors and has been the primary target for anti-angiogenic therapy of cancer [33]. Pro-angiogenic growth factors can anergize the tumor vessels by reducing expression of adhesion molecules and thereby suppress infiltration of T-lymphocytes [14, 34, 35]. In line with this, several reports have shown benefits of repressing VEGF-signaling in the tumor microenvironment during immunotherapy. Antibody targeting of VEGF/VEGFR2 signaling increases the efficacy of adoptive T-cell therapy of established B16 melanomas [36] and has been demonstrated to enhance the antitumor effect of agonistic CD40-antibodies after subcutaneous injection of RM-1 prostate cancer cells [37].

It was recently shown that increased hypoxia induces MDSC accumulation in B16.F10 tumors in pericyte deficient mice [38]. In contrast, the reduced accumulation of MDSCs noted in tumors treated with anti-CD40 mAb and sunitinib in our study was not related to hypoxia, tumor vessel pericyte coverage or vessel functionality since these parameters were similar in all treatment groups (Figure 2E–2F, Supplementary Figure S2). Mo-MDSCs and Gr-MDSCs isolated from B16.F10 tumors express VEGFR1 and VEGFR2, suggesting that sunitinib may affect MDSC accumulation in the tumor microenvironment by directly targeting VEGFR-signaling in MDSCs (Supplementary Figure S3E-S3F). However, it is also possible that sunitinib acts through inhibition of non-VEGF-induced signaling pathways. Sunitinib appears to be more effective than other anti-VEGF strategies at reducing MDSCs in experimental breast cancer models [39]. Similarly, MDSCs were not reduced when treating RCC patients with an anti-VEGF antibody, while another study showed that sunitinib therapy was able to reverse MDSC accumulation in RCC [40, 41]. This indicates that co-treatment with sunitinib may be superior to anti-VEGF antibody therapy with respect to efficiency in reducing immunosuppressive cells in the tumor.

While the main antitumor effects of agonistic anti-CD40 antibodies are through activation of DCs, sunitinib has been shown to decrease DC activation [20, 21]. However, CD40-induced DC activation was not hampered by co-treatment with sunitinib in neither the B16.F10 melanoma model nor the T241 fibrosarcoma model (Figure 2A-2B). Our study shows that the combination with sunitinib enables the CD40-induced DC activation while reducing accumulation of MDSCs. In addition, we find that anti-CD40 antibodies and sunitinib therapy up-regulate ICAM-1 in B16.F10 melanoma and VCAM-1 in both models, thereby decreasing the endothelial barrier to lymphocyte recruitment (Figure 4A-4D). Consequently, we find high numbers of intratumoral CD8^+^ T-lymphocytes after treatment with anti-CD40 antibodies in combination with sunitinib, associated with a significant reduction in tumor growth and increased survival of mice bearing either B16.F10 melanoma or T241 fibrosarcoma (Figure 1A-1D, Figure 4E-4F).

CD40-activating immunotherapy is currently in multiple clinical trials, either as monotherapy or in combination with other therapies [NCT02379741, NCT02482168, NCT02376699, 2-4]. Despite promising results, side effects such as cytokine release syndrome and hepatotoxicity associated with a high-dose administration of agonistic CD40 mAb may limit efficacy and even result in lethality when combined with chemotherapy [42]. Our results indicate that combining a local low-dose administration of agonistic CD40-antibodies with sunitinib would increase the efficacy of the therapy by reducing immunosuppressive cells and relieving endothelial anergy, thereby facilitating infiltration and activation of cytotoxic T-cells. This may have important implications for the use of CD40-activating immunotherapy in the clinic, warranting further studies in patient cohorts.

## MATERIALS AND METHODS

### Cell culture

Murine B16.F10 melanoma and T241 fibrosarcoma cells (American Type Culture Collection, Manassas, VA, USA via LGC Standards) were cultured in Dulbecco's Modified Eagle Medium (DMEM) supplemented with GlutaMAX™ (Life Technologies, carlsbad, CA, USA) and 10% fetal calf serum (FCS) (Sigma-Aldrich, MO, St. Louis, USA) at 37°C and 5% CO_2_ in a humidified cell incubator. The cell lines were not authenticated after purchase but were routinely tested negative for mycoplasma contamination using the MycoAlert Detection Kit (Lonza, Basel, Switzerland).

### Experimental tumor models

Male C57BL/6 wild-type mice, 7-8 weeks old, were purchased from Taconic M&B (Bomholt, Denmark) and received 2.5×10^5^ B16.F10 or T241 cells subcutaneously on the right flank on day 0. The treatment regimens included 30 μg agonistic rat-anti-mouse CD40 antibody (clone: FGK4.5) (Bio X Cell, USA) in phosphate-buffered saline (PBS) (Life Technologies, carlsbad, CA, USA) or PBS injected peritumorally and sunitinib (LC Laboratories, Woburn, MA, USA) at a dose of 50 mg/kg or vehicle (carboxymethylcellulose sodium 0.5% wt/vol, NaCl 1.8% wt/vol, Tween-80 0.4% wt/vol, benzyl alcohol 0.9% wt/vol, and H_2_O, pH 6.0) administered via oral gavage. On day 10 for the B16.F10 and on day 12 for the T241 studies mice had palpable tumors and were randomized into 4 treatment groups. In the endpoint experiments the mice were treated with two anti-CD40 or PBS injections with 2 days' interval and sunitinib or vehicle for 4 consecutive days. In the survival experiments, tumors were treated three times (T241 model) or two times (B16.F10 model) with anti-CD40 or PBS injections with a 2 days' interval. Sunitinib or vehicle was administered for four consecutive days for the B16.F10 model or seven consecutive days for the T241 model.

For the B16.F10 melanoma model, a survival experiment with continued treatments was performed. Once the mice had palpable tumors (day 10 after tumor cell injection) they were randomly divided and treated with anti-CD40 (90 μg) or PBS every third day and sunitinib or vehicle on a daily basis for 35 consecutive days. Throughout the animal experiments the tumors were measured by caliper at least every other day and tumor size was calculated by the ellipsoid formula: 4/3 × π × a (radius of length) × b (radius of width) × c (radius of depth).

In the endpoint experiments, the mice were sacrificed on day 14 for B16.F10 and day 16 for T241, one day after the last treatment occasion. Tumor draining lymph nodes were collected for flow cytometry analysis, while the tumors were dissected and either snap frozen in isopentane/dry ice or further processed for flow cytometry. To investigate tumor vessel perfusion, 100 μl of 1 mg/ml biotin-labeled lycopersicon esculentum (tomato) lectin (Vector Laboratories, Burlingame, CA, USA) was injected in the orbital plexus of mice. The lectin was allowed to circulate for 5 minutes. To detect hypoxia, 1.5 mg/100 μl PBS of pimonidazole hydrochloride (Hypoxyprobe-1, Hypoxyprobe Inc., Massachusetts, USA) was injected intraperitoneally and circulated for 1-1.5 hours. The mice were then sedated with ketamine/xylazine and perfused with PBS followed by 4% PFA through the heart. The tumors were dissected, fixed in 4% PFA overnight and cryoprotected in 30% sucrose/PBS, after which they were embedded in OCT Cryomount (Histolabs, Göteborg, Sweden). In the survival studies, the mice were sacrificed when the tumors had a volume ≥ 1 cm^3^ or when ulcers developed.

All animal experiments, approved by a regional Ethics Committee (ethical permit C147/12 and C1/14), were executed according to the guidelines for animal experimentation and welfare of Uppsala University.

### Flow cytometry

Tumors enzymatically digested by ± 2.3 Wunsch units / ml Liberase TL (Roche, Basel, Switzerland) and tumor draining lymph nodes were passed through 70 μm cell strainers. The generated single cell suspensions were stained with the live/dead marker Zombie Aqua (Biolegend, San Diego, CA, USA) and subsequently blocked for unspecific binding to CD16/32 (TruStain fcX, Biolegend). In order to investigate different myeloid cell populations and endothelial cell activation the following antibodies were diluted in FACS buffer (1% FCS, 0.02% NaN_3_ and 3 mM EDTA in PBS): anti-CD11b (clone M1/70, Biolegend), anti-CD11c (clone N418, Biolegend), anti-B220 (clone RA3-6B2, Biolegend), anti-CD86 (clone GL-1, Biolegend), anti-CD45 (clone 30-F11, Biolegend), anti-Gr1 (clone RB6-8CS, Biolegend), anti-Ly6C (clone HK1.4, Biolegend), Ly6G (clone 1A8, Biolegend), ICAM-1 (clone YN1/1.7.4, Biolegend), VCAM-1 (clone MVCAM.A, Biolegend) and CD31 (clone MEC 13.3, BD Biosciences, Franklin Lakes, NJ, USA). Samples were washed with FACS-buffer and analyzed in a FACSCanto II cytometer (BD Biosciences, Franklin Lakes, NJ, USA). Data analysis was performed with FlowJo software (TreeStar, Ashland, OR, USA).

### FACS sorting, RNA extraction and cDNA preparation

B16.F10 melanoma tumors from mice treated with sunitinib and anti-CD40 (combination therapy group) or vehicle and PBS (control group) were digested with 5 ml/g of DMEM supplemented with 5 mg/mL Collagenase II and 50 μg/mL DNase I (both from Sigma-Aldrich, MO, St. Louis, USA) for 40 min at 37°C in a shaking incubation. The tumor lysates were passed through a 70 μm cell strainer and erythrocyte lysis was performed on ice. The samples were blocked with Fc block and stained with antibodies (Biolegend, San Diego, CA, USA) against B220 (clone RA3-6B2), CD11b (clone M1/70), CD11c (clone N418), Ly6C (clone HK1.4), and Ly6G (clone 1A8) for 40 minutes. After washing with FACS sorting buffer (2% BSA and 1mM EDTA in PBS) the samples were stained with DAPI and sorted in a FACSaria III (BD, Biosciences, Franklin Lakes, NJ, USA). The monocytic (B220^−^CD11b^+^Ly6G^lo^Ly6C^+^) and granulocytic (B220^−^CD11b^+^Ly6G^+^Ly6C^+^) MDSC subsets were sorted and collected in PBS, centrifuged and RNA was directly extracted by using the miRNeasy Micro kit (Qiagen, Hilden, Germany). cDNA was generated using the SuperScript III kit (Life Technologies, Carlsbad, CA, USA).

### Gene expression analysis by TaqMan quantitative real-time PCR

Quantitative real-time PCR was performed using TaqMan Universal PCR Master Mix and probes (Life Technologies, Carlsbad, CA, USA) to determine the expression levels of known immunosuppressive genes in the FACS-isolated MDSC subsets. The following probes were used: Arginase-1 (*Arg1*), Mm00475991_m1, iNOS (*Nos-2*), Mm00440502_m1, p47phox (*Ncf-1*), Mm00447921_m1, gp91phox (*Cybb*), Mm01287742_m1, Actb (*Actb*), Mm00607939_s1. Each reaction was run using the Standard Mode conditions (StepOne Plus Real-Time PCR System, Applied Biosystems, Foster City, CA, USA) in technical duplicates and biological triplicates. Relative expression (RE) was determined with the formula RE _gene x_ = 2^-(Cq Actb-Cq gene x)^.

### Immunofluorescence staining and image analysis

Cryosections (7 μm-10 μm) from snap frozen tumors were fixed with ice-cold acetone for 15 min and blocked with 3% bovine serum albumin (BSA) in PBS for 1 h at room temperature. The sections were incubated overnight at 4°C with antibodies against CD31 (clone 2H8, Thermo Scientific, Waltham, Massachusetts, USA) to detect the vasculature, Ki-67 (polyclonal, Santa-Cruz, Dallas, Texas, USA), streptavidin-Alexa-488 (Life Technologies, Carlsbad, CA, USA) to detect biotin-labeled lycopersicon esculentum (tomato) lectin, DylightTM594-conjugated antibodies (Hypoxyprobe Inc., Massachusetts, USA) that bind to Hypoxyprobe-1 (pimonidazole) adducts in hypoxic tissues and APC-conjugated antibodies against CD8 (clone 53-6.7, Biolegend, San Diego, CA, USA) to detect CD8^+^ T-cells. After washing with PBS 3 times, sections were incubated with Cy3- or Alexa-647- conjugated goat anti-hamster secondary antibodies (Jackson ImmunoResearch, West Grove, PA, USA) in blocking solution for 1 h at room temperature. For Ki-67 staining the secondary antibody was Alexa-488 donkey anti-goat (Life Technologies, Carlsbad, CA, USA). Sections were then counterstained with Hoechst 33342 (Sigma-Aldrich, MO, St. Louis, USA) and mounted with Fluoromount-G (Southern Biotechnology, Birmingham, AL, USA). Tile-scan images (10x magnification for CD31, and 20x for lectin, hypoxia and CD8 stainings) from whole tumor sections were taken on a Zeiss Axioimager microscope using the ZEN Blue software (Zeiss, Oberkochen, Germany). For Ki67 and CD8 co-localization 5 optical fields with 40x magnification were acquired per tumor section on a Leica DMi8 inverted micrsoscope (Leica Microsystems, Wetzlar, Germany). Image J software was used for staining quantification for vessel density (ratio of CD31 positive area to whole tumor section area), vessel perfusion (ratio of lectin perfused to total CD31^+^ vessels), Hypoxyprobe-1 (ratio of hypoxic surface area to whole tumor section area) and manual cell counting for CD8^+^ T-cells as well as CD8^+^Ki67^+^ excluding the core due to necrotic areas.

### Statistical analysis

Statistical analysis was performed using the GraphPad Prism 6.0 (GraphPad, La Jolla, CA, USA) software. Kaplan-Meier survival curves were analyzed using the Gehan-Breslow-Wilcoxon test. A student's t-test (unpaired, two-tailed) or a one-way ANOVA (Fisher's LSD test) was performed for to determine statistically significant differences between groups while interaction between agonistic CD40-antibody treatment and sunitinib was tested using a two-way ANOVA.

## SUPPLEMENTARY MATERIALS FIGURES


