# Supplementary material for: Sunitinib enhances the antitumor responses of agonistic CD40-antibody by reducing MDSCs and synergistically improving endothelial activation and T-cell recruitment
Source: Oncotarget. 2016 Jul 1;7(31):50277–89. doi: 10.18632/oncotarget.10364 (PMC5226582; doi:10.18632/oncotarget.10364)
Supplement: Supplementary file 1 [file oncotarget-07-50277-s001.pdf]

Sunitinib enhances the antitumor responses of agonistic CD40-antibody by reducing MDSCs and synergistically improving endothelial activation and T-cell recruitment

SUPPLEMENTARY MATERIALS AND METHODS

Gene expression analysis by quantitative SYBR Green real-time PCR

To determine the VEGF and CD40 expression levels in B16.F10 and T241 tumor tissues (Supplementary Figure S2), RNA was extracted (from 30 cryosections/sample, 10 µm thick each) and cDNA was prepared from control tumor tissues. To determine the VEGFR1 and VEGFR2 expression in B16.F10 tumor-derived MDCS, cells were isolated by FACS-sorting and cDNA was prepared as described in the "Materials and Methods" section. Quantitative PCR was performed using SYBR Green (Life Technologies, Carlsbad, CA, USA) in technical duplicates and biological triplicates (Mx3000P QPCR system, Agilent Technologies, Santa Clara, California, USA). Murine (m) hypoxanthine-guanine phosphoribosyltransferase (HPRT) housekeeping gene was used as reference gene. Primer sequences (Life Technologies, Carlsbad, CA, USA) were the following:

mHPRT forward (fwd): 5'CAAACTTTGCTTTCCC TGGT-3',

mHPRT reverse (rs): 5'-TCGAGAGGTCCTTTT CACC-3',

mVEGFA forward (fwd): 5'- AAGGAGAGCAG AAGTCCCATGA-3',

mVEGFA reverse (rs): 5'-CTCAATTGGACGG CAGTAGCT-3',

mCD40 forward (fwd): 5'-GCCATCGTGGAGG TACTGTT-3',

mCD40 reverse (rs): 5'-CTGCGATGGTGTCTT TGCCT-3'.

mVEGFR1 forward (fwd): 5'-TGCCCTATGAT GCCAGCA-3'

mVEGFR1 reverse (rs): 5'-TTCCGAGCGATTTG CCTAGT-3'

mVEGFR2 forward (fwd): 5'- ACAGACCCGGCC AAACAA-3'

mVEGFR2 reverse (rs): 5'-TTCCCCCCTGGAA ATCCTC -3'

Relative expression (RE) was determined with the formula RE gene x= 2-(Cq Hprt-Cq gene x).

Pericyte coverage and granzyme B immunofluorescence staining

Cryosections (10µm) from snap frozen tumors with representative sizes per treatment group from endpoint experiments were fixed with ice-cold acetone for 15 min and blocked with 3% bovine serum albumin (BSA) in PBS for 1 h at room temperature. The sections were incubated overnight at 4°C with antibodies against CD31 (clone 2H8, Thermo Scientific, Waltham, Massachusetts, USA) to detect the vasculature and desmin (polyclonal rabbit anti-mouse, Abcam, Cambridge, UK) for pericyte staining. After washing with PBS 3 times, sections were incubated with Alexa-647-conjugated goat anti-hamster secondary antibodies (Jackson ImmunoResearch, West Grove, PA, USA) and Alexa-568-conjugated goat anti-rabbit (Abcam, Cambridge, UK) in blocking solution for 1 h at room temperature. To detect granzyme B producing CD8 T-cells, the sections were incubated overnight at 4°C with antibodies against granzyme B (GrzB) (clone GB11, Biolegend, San Diego, CA, USA) directly conjugated with Alexa-647 and CD8 (clone 53-6.7, BD Biosciences, Franklin Lakes, NJ, USA). After washing with PBS 3 times, sections were incubated with Alexa-594-conjugated donkey anti-rat (ThermoFisher, Massachusetts, USA) in blocking solution for 1 h at room temperature. Sections were then counterstained with Hoechst 33342 (Sigma-Aldrich, MO, St. Louis, USA) and mounted with Fluoromount-G (Southern Biotechnology, Birmingham, AL, USA). Tile-scan images (10x magnification for CD31/desmin staining and 20x for granzyme B/CD8 staining) from whole tumor sections were taken on a Zeiss Axioimager microscope using the ZEN Blue software. Cell Profiler software (Zeiss, Oberkochen, Germany) was used for quantifying the desmin positive area around the CD31 positive vessel area and Image J for manual cell counting of $CD8^{+}GrzB^{+}$ cells excluding the core due to necrotic areas.

SUPPLEMENTARY FIGURES

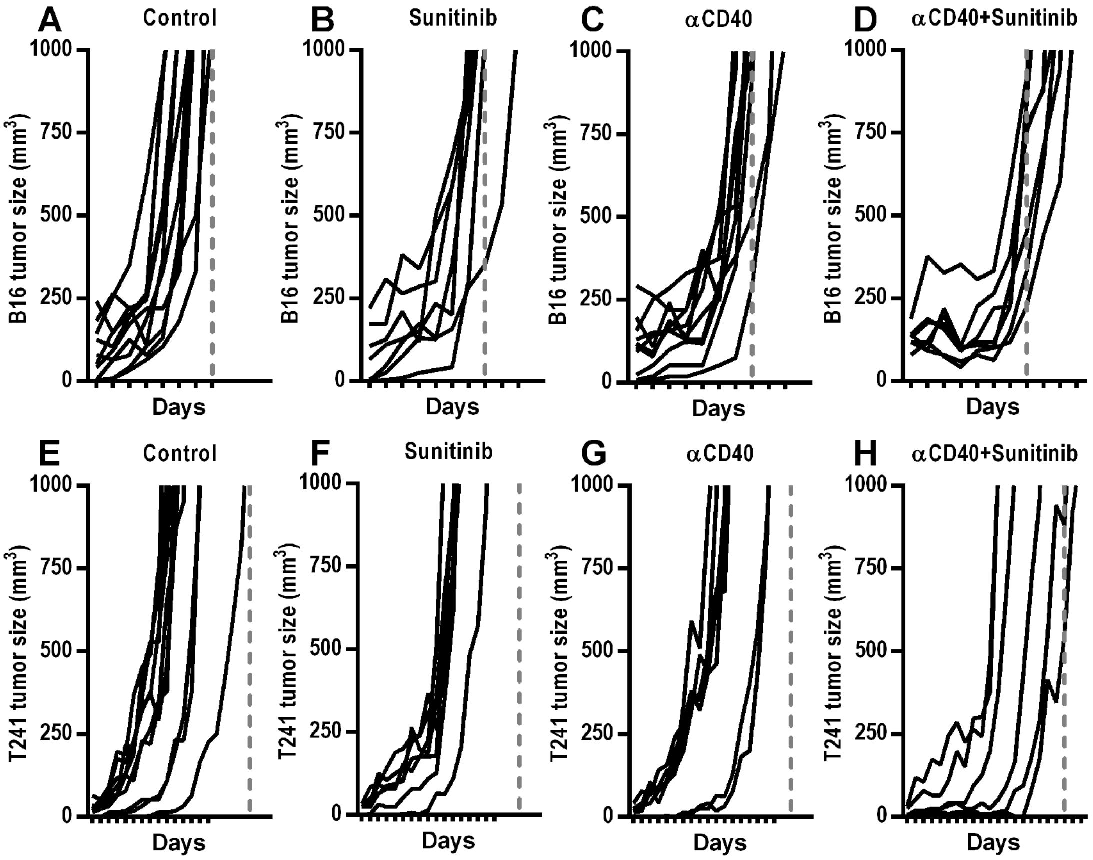

Supplementary Figure S1: Tumor growth of mice with B16.F10 melanoma or T241 fibrosarcoma tumors treated with CD40 mAb and/or sunitinib A-D. Individual tumor growth curves in mice bearing B16.F10 tumors of the control group (A), or treated with either sunitinib (B) or CD40 mAb (C) monotherapies or with CD40 mAb and sunitinib combination (D) therapy. Data show representative tumor growth curves from one out of five independent experiments. **E-H.** Individual tumor growth curves in mice bearing T241 tumors of the control group (E), or treated with either sunitinib (F) or CD40 mAb (G) monotherapies or with CD40 mAb and sunitinib combination (H) therapy. Data show representative tumor growth curves from one out of three independent experiments. Dashed lines indicate the day that the last control-treated mouse was sacrificed due to tumor size ≥1cm³.

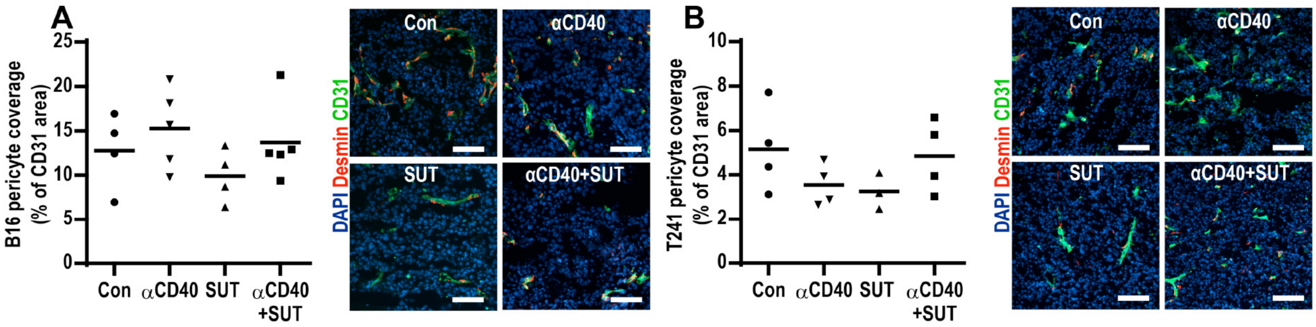

Supplementary Figure S2: CD40 mAb and sunitinib combination therapy does not affect pericyte coverage in B16.F10 or T241 tumor vessels. A-B. Pericyte coverage of tumor vessels in B16.F10 (A) and T241 (B) tumors as analyzed by immunofluorescence staining. Quantifications of desmin$^+$ pericytearea (red) surrounding CD31$^+$ endothelial cells (green) were performed using CellProfiler by analyzing tile scans covering entire tumor sections (10x). Data points represent analysis of tumors in different mice one day after the last treatment. Images are representative for each treatment group (10x, scale bar: 100 µm). Nuclear staining by Hoechst 33342 (blue).

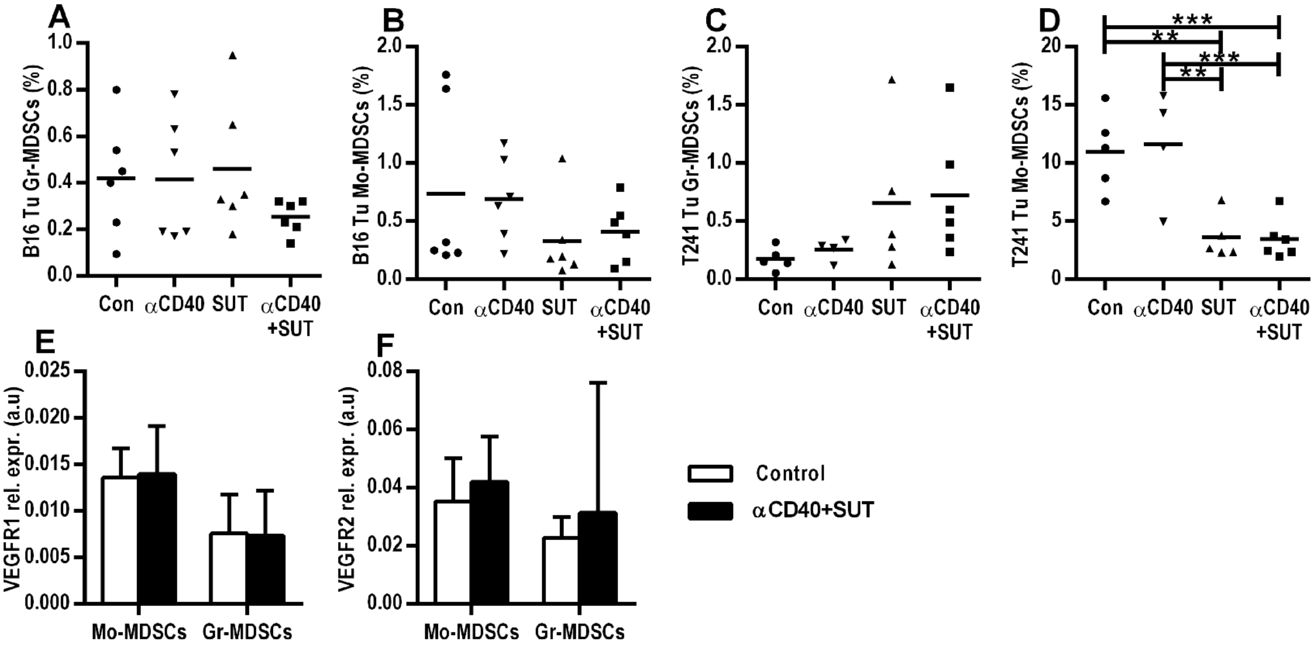

Supplementary Figure S3: Effects of CD40 mAb therapy in combination with sunitinib in CD11b$^+$Gr1$^+$ myeloid-derived suppressor cells in B16.F10 melanoma and T241 fibrosarcoma tumors A-B. FACS analysis of the granulocytic (A) and the monocytic (B) CD11b$^+$Gr1$^+$ myeloid-derived suppressor cell subsets in B16.F10 melanoma tumors treated with anti-CD40 mAbs and/or sunitinib. **C-D.** FACS analysis of the granulocytic (C) and the monocytic (D) CD11b$^+$Gr1$^+$ myeloid-derived suppressor cell subsets in T241 fibrosarcoma tumors treated with anti-CD40 mAbs and/or sunitinib. Data points represent analysis of tumors in different mice one day after the last treatment. **E-F.** Gene expression of VEGFR1 (E) and VEGFR2 (F) in FACS-sorted monocytic and granulocytic MDSCs from B16.F10 tumors. Values depict the relative gene expression levels of VEGFR1 and VEGFR2 to the HPRT reference gene as analyzed by SYBR green quantitative PCR. (n=3 tumors from different mice, mean, SD). FACS-sorting was performed two days after the last treatment.

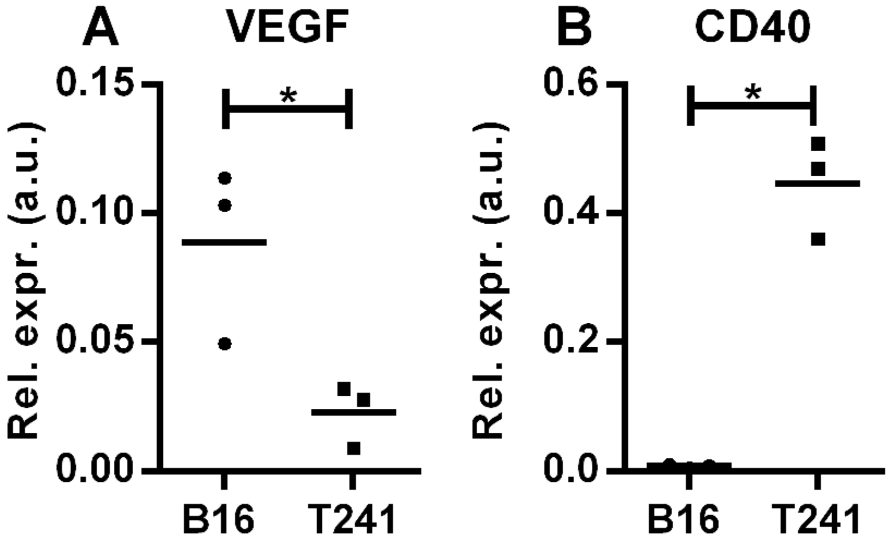

Supplementary Figure S4: Expression of VEGF and CD40 in B16.F10 and T241 tumor tissues. Values depict the relative gene expression levels of VEGF **A.** and CD40 **B.** to HPRT reference gene in B16.F10 and T241 control tumor tissues (n=3 tumors from different mice, mean, **p* < 0.05, unpaired Student's t-test).

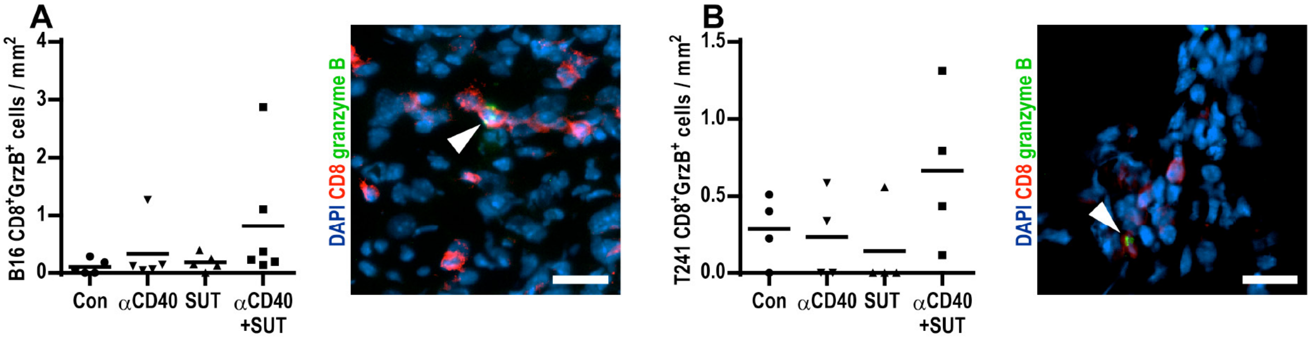

Supplementary Figure S5: Granzyme B expression by $CD8^+$ T cells in B16.F10 and T241 tumors A-B. Quantification of $CD8^+GrzB^+$ T cells/mm^2 in tumor tissue excluding necrotic areas in B16.F10 (A) and T241 (B) tumors (data points show analysis of total tumor area tile scans, 20x, mean, one-way ANOVA). Images show representative stainings of tumors treated with anti-CD40 mAb in combination with sunitinib with granzyme B^+ in green, $CD8^+$ in red and Hoechst 33342 in blue (20x, scale bar: 20 µm). Data points indicate analysis of tumors in different mice one day after the last treatment.
